# Multimorbidity and quality of primary care after release from prison: a prospective data-linkage cohort study

**DOI:** 10.1186/s12913-022-08209-6

**Published:** 2022-07-07

**Authors:** Lucas Calais-Ferreira, Amanda Butler, Stephan Dent, David B. Preen, Jesse T. Young, Stuart A. Kinner

**Affiliations:** 1 Justice Health Unit, Centre for Health Equity, Melbourne School of Population and Global Health, The University of Melbourne, Level 3, 207 Bouverie St, Carlton, Melbourne, Victoria 3070 Australia; 2 Centre for Adolescent Health, Murdoch Children’s Research Institute, Level 3, 207 Bouverie St, Carlton, Melbourne, Victoria 3070 Australia; 3 Centre for Epidemiology and Biostatistics, Melbourne School of Population and Global Health, The University of Melbourne, Level 3, 207 Bouverie St, Carlton, Melbourne, Victoria 3070 Australia; 4 Department of Family Medicine, McMaster University, Hamilton, Ontario Canada; 5 Faculty of Medicine, Dentistry and Health Sciences, The University of Melbourne, Melbourne, Victoria Australia; 6 Centre for Health Services Research, School of Population and Global Health, The University of Western Australia, Perth, Western Australia Australia; 7 National Drug Research Institute, Curtin University, Perth, Western Australia Australia; 8 School of Population Health, Curtin University, Perth, Western Australia Australia; 9 Griffith Criminology Institute, Griffith University, Brisbane, Queensland Australia

**Keywords:** Prisons, Morbidity, Quality of care, Primary care, Social determinants of health, Administrative data, Data linkage

## Abstract

**Background:**

The period after release from prison can be challenging, especially due to a higher risk of morbidity and mortality despite commonly increased use of healthcare services. However, little is known about the quality of the healthcare offered to this population, which limits the possibility of addressing this important health inequity. This study characterised multimorbidity and investigated the relationship between multimorbidity and quality of primary healthcare in adults within 2 years after release from prison.

**Methods:**

This was a prospective cohort study of 1046 participants of a service brokerage intervention after release from prison between August 2008 and July 2010 in Queensland, Australia. Participants had their baseline survey and clinical data linked prospectively with their medical, correctional and death records. Multimorbidity was ascertained using the Cumulative Illness Rating Scale and classified into three categories: none, moderate (morbidity in 2–3 domains) and complex (morbidity in 4 or more domains). Outcomes were Usual Provider Continuity Index (UPCI), Continuity of Care (COC) Index, and having at least one extended primary care consultation (> 20 minutes). Descriptive statistics and logistic regression were used in the analyses.

**Results:**

Multimorbidity was present for 761 (73%) participants, being more prevalent among females (85%) than males (69%), *p* < 0.001. Moderate multimorbidity was not associated with UPCI or COC, but was associated with having at least one long consultation (AOR = 1.64; 95% CI:1.14–2.39), after adjusting for covariates. Complex multimorbidity was positively associated with all outcomes in the adjusted models. Indigenous status was negatively associated with UPCI (AOR = 0.54; 95% CI: 0.37–0.80) and COC (AOR = 0.53; 95% CI: 0.36–0.77), and people younger than 25 years were at 36% lower odds (AOR = 0.64; 95% CI: 0.44–0.93) of having a long consultation than the middle-aged group (25–44 years) in the adjusted models.

**Conclusion:**

Moderate multimorbidity was associated with having at least one extended primary care consultation, but not with adequate continuity of care, for adults within 2 years of being released from prison. Nearly half of those with complex multimorbidity did not receive adequate continuity of care. The quality of primary care is inadequate for a large proportion of adults released from prison, constituting an important and actionable health inequity.

**Supplementary Information:**

The online version contains supplementary material available at 10.1186/s12913-022-08209-6.

## Introduction

People in prison often have complex healthcare needs associated with a high prevalence of mental illness, substance use problems, cognitive disability, communicable and non-communicable disease, and social disadvantage [1, 2]. Chronic health conditions frequently co-occur such that multimorbidity (the presence of more than one chronic disease in an individual) is common [3]. However, multimorbidity in prisons has not been well characterised, and the prevalence of multimorbidity among people in prisons is unknown.

The period immediately following release from prison is associated with increased risk of preventable morbidity and mortality, including suicide and self-harm, drug overdose, decompensation of disease, and preventable hospitalisation [4–6]. Although the rate of primary care contact in people released from prison is higher than that of the general population [7, 8] continuity of care is a challenge particularly because people in prison are excluded from federally subsidised medical care (Medicare) [1]. In the United States, recently expanded eligibility for Medicaid and pre-release enrollment assistance have increased access to primary care for adults released from prison [9].

High-quality primary care has been shown to predict better health outcomes in the general population, including reduced hospitalisation, emergency department attendance, and mortality; and increased preventive healthcare, patient satisfaction, and efficiency of chronic disease management [10, 11]. In particular, continuity of care has been linked to decreased hospitalisation rates for multimorbid patients [12]. Therefore, the aims of this study were to (a) characterise multimorbidity, and (b) examine the relationship between multimorbidity in prison and quality of primary healthcare within 2 years after release from prison.

## Methods

This study involved secondary analysis of data from a randomised controlled trial of a service brokerage intervention for people released from prison, which has been described previously [13]. In-prison survey data and coded prison medical records were linked prospectively with their records from Medicare (Australia’s universal health insurance scheme), the National Death Index (NDI), and correctional services. All participants provided written informed consent. This study is reported in accordance with the RECORD Statement [14].

### Study population

Participants (*N* = 1046) were adults interviewed within 6 weeks of expected release from seven prisons in the state of Queensland, Australia, between August 2008 and July 2010. Participants were followed from prison release date for 2 years, or until death if they died within this period. The cohort was representative of people released from prisons in Australia, on criminal justice and sociodemographic measures, except that women were intentionally oversampled.

### Baseline measures

Self-reported measures at baseline included age, sex, Indigenous status, employment status, years of schooling, accommodation status, relationship status, transitional program participation, history of injecting drug use, smoking status, and years of tobacco smoking. Validated screening tools administered at baseline included the Hayes Ability Screening Index [15] (HASI - a measure of intellectual disability), Patient Activation Measure [16] (PAM - a measure of confidence in self-managing health conditions), and Enriched Social Support Inventory [17] (ESSI - a measure of perceived social support).

### Ascertainment and definition of multimorbidity

All episodes of healthcare contact during the index incarceration, recorded in prison medical records, were coded using the International Classification of Primary Care, Second Edition (ICPC-2). These data were subsequently coded by two trained researchers using the Cumulative Illness Rating Scale (CIRS), a validated tool for measuring multimorbidity [18].

The CIRS uses a scoring system to classify chronic illnesses into 14 distinct anatomical domains, and assigns each domain a severity rating of zero to four where 0 = no problems; 1 = mild or past serious problems; 2 = moderate problems with minor impact on morbidity; 3 = severe chronic problems that cause significant morbidity; and 4 = extreme severe functional impairment.

The basis for our scoring system was Miller and Hudon’s CIRS-G manual [19], which was initially developed for a geriatric primary care setting and contains specific rules for each domain. We created additional coding rules and amended some existing coding rules to better capture illnesses that are prevalent in this young, disadvantaged population, primarily regarding coding of severity in the psychiatric and musculoskeletal domains (see Table S1).

A score of 1 or more in a domain was considered indicative of morbidity. To ascertain multimorbidity, we computed a count of domains that were positive for morbidity and participants were then sorted into three mutually exclusive categories: no multimorbidity (morbidity in 0–1 domains), moderate multimorbidity (morbidity in 2–3 domains), and complex multimorbidity (morbidity in 4 or more domains).

### Quality of primary healthcare

Primary care encounters after release from prison were identified using Medicare item numbers (see Table S2 for details). Services provided by primary care doctors and on their behalf by nurse practitioners were defined as primary care encounters. Measures of quality of primary healthcare included (a) continuity of care, and (b) use of extended consultations. Item numbers were sorted by length of consultation into standard (< 20 minutes; Level A and B consultations) and extended (≥20 minutes; Level C and D consultations, Aboriginal Health Check, Primary Care Management Plan, Primary Care Mental Health Plan). To produce a conservative estimate of extended consultation prevalence, Medicare item numbers with unspecified length (11.5%) were coded as standard length. Time spent in prison during reincarceration was excluded from follow-up time, as Medicare-funded services are not available for people in prison in Australia [20]. A high rate of primary care access was defined as nine or more consultations per person-year, representing the fourth quartile (top 25%) of participants with regard to their number of primary care contacts.

Continuity of care was measured using two common indices of continuity of care: the Usual Provider Continuity Index [21] (UPCI) and the index of Continuity of Care [22] (COC). The UPCI gives a measure of the proportion of encounters that a patient has with their most visited doctor. Consistent with the literature [21], a UPCI of 0.5 or higher, indicating that a patient visited the same primary care doctor at least half the time, was considered adequate. The COC weights both the frequency of visits to each provider and the dispersion of visits between providers. Index values range from 0 (i.e., each visit made to a different physician) to 1 (i.e., all visits made to the same physician). Adequate COC was defined as > 0.25 (the median COC in this cohort). Patients with only one primary care contact during follow-up (*n* = 202) were excluded from the continuity of care analyses.

### Data analysis

Descriptive statistics were calculated for all measures. Unadjusted rates of primary care contact post-release were calculated per person-year, deducting time spent in prison from person-time, and censoring at death or 2 years after release. Logistic regression was used to examine the association between multimorbidity (no multimorbidity, moderate, complex), rate of primary care contact, and quality of care (COC and UPCI, at least one extended consultation), adjusting for social determinants of health (age, sex, Indigenous status, unstable accommodation, unemployment, < 10 years of schooling) and history of incarceration (juvenile and adult).

We undertook four sensitivity analyses (see Table S3). First, we restricted the exposure to physical multimorbidity to test whether any association between multimorbidity and quality of primary care was driven by psychiatric comorbidity. Second, we excluded records of primary care encounters with unspecified length to assess bias from differences in quality of data in medical records, especially in the association between multimorbidity and long consultations. Third, we excluded participants who were reincarcerated during follow-up to test the assumption that quality of care is unrelated to episodes of reincarceration. Finally, we re-included those with only one primary care consultation during follow-up to assess potential changes in the association between multimorbidity and long consultations. All data analysis was performed using Stata v15.1 [23].

## Results

Characteristics of the cohort at baseline, according to sex, are summarised in Table 1. During follow-up, 21 of 1046 participants (2.0%) died, and 457 (43.7%) were reincarcerated at least once. Over 2 years of follow-up, after censoring for deaths and excluding follow-up time in prison, the mean follow-up time in the community was 655 days (SD = 143.3).

**Table 1:** Characteristics of the cohort at baseline

	Total (*n* = 1046)	Males (*n* = 792)	Females (*n* = 254)	*p*-value
Age in years				0.001
< 25	256/1046 (24.5%)	195/792 (24.6%)	61/254 (24.0%)	
25–44	631/1046 (60.3%)	459/792 (58.0%)	172/254 (67.7%)	
45+	159/1046 (15.2%)	138/792 (17.4%)	21/254 (8.3%)	
Indigenous	251/1046 (24.0%)	166/792 (21.0%)	85/254 (33.5%)	< 0.001
Unemployed ^a^	502/1045 (48.0%)	355/791 (44.9%)	147/254 (57.9%)	< 0.001
< 10 years of schooling	448/1043 (43.0%)	356/790 (45.1%)	92/253 (36.4%)	0.015
Unstable accommodation ^a^	179/1041 (17.2%)	139/787 (17.7%)	40/254 (15.8%)	0.482
Not in a stable relationship	603/1038 (58.1%)	465/786 (59.2%)	138/252 (54.8%)	0.218
Transitional program participation	166/1046 (15.9%)	131/792 (16.5%)	35/254 (13.8%)	0.295
Ever injected drugs	580/1045 (55.5%)	421/791 (53.2%)	159/254 (62.6%)	0.009
Below cohort median level of social support (ESSI) ^b^	490/1042 (47.0%)	386/790 (48.9%)	104/252 (41.3%)	0.036
Below cohort median level of patient activation (PAM) ^b^	510/1042 (48.9%)	381/789 (48.3%)	129/253 (51.0%)	0.455
Screened as potentially having an intellectual disability (HASI)	238/1021 (23.3%)	211/770 (27.4%)	27/251 (10.8%)	< 0.001
Socioeconomic status of residential area in most disadvantage quintile ^c^	249/1026 (24.3%)	183/773 (23.6%)	66/249 (26.5%)	0.344
Released on parole	389/1046 (37.2%)	293/792 (37.0%)	96/254 (37.8%)	0.818
Prior adult incarceration	688/1044 (65.9%)	515/791 (65.1%)	173/253 (68.4%)	0.339
History of juvenile detention	290/1046 (27.7%)	239/792 (30.2%)	51/254 (20.1%)	0.002
History of traumatic brain injury or lead poisoning	78/1046 (7.5%)	61/792 (7.7%)	17/254 (6.7%)	0.594
Received service intervention (Passports)	527/1046 (50.4%)	397/792 (50.1%)	130/254 (51.2%)	0.770

^a^prior to prison

^b^PAM median score was 63.2

^c^using release postcode and 2011 Socio-Economic Indexes for Areas (SEIFA) statistics; Note: denominators are included to indicate missing data

### Multimorbidity

Of 1046 cohort participants, 943 (90.2%) had at least one chronic illness, and 761 (72.8%) were multimorbid (464 [44.4%] with moderate and 297 [28.4%] with complex multimorbidity). Multimorbidity was more common for females (216/254, 85.0%) than for males (545/792, 68.8%), *p* < 0.001, and for participants aged ≥45 years (134/159, 84.3%) than for younger groups, *p* < 0.001. After excluding psychiatric illness, 298 (28.5%) participants remained multimorbid. More than half of those with a (non-drug and alcohol-related) psychiatric illness also had a substance use disorder and a chronic physical illness (59/94; 62.7%). Nearly all participants with a psychiatric illness were multimorbid (584/645, 90.6%).

### Quantity and quality of primary care consultations

The rate of primary care visits per person-year, after excluding time in prison and censoring for deaths, was 7.33 (95%CI 7.20–7.45) visits per person-year. More than three-quarters of the 1046 participants had at least one primary care visit during follow-up (914, 87.4%), and 620 (59.3%) had at least one extended consultation. Four in five participants (844, 80.7%) had two or more primary care encounters during follow-up.

Women were more likely than men (191/233, 82.0% vs. 414/611, 67.8%) to have received an extended consultation (*p* < 0.001). Table 2 describes the psychiatric conditions, multimorbidity, and quality of primary care in the cohort, stratified by sex.

**Table 2:** Prevalence of psychiatric conditions, multimorbidity and quality of primary care, according to sex

	Total (*n* = 1046)	Males (*n* = 792)	Females (*n* = 254)	*p*-value ^e^
Any psychiatric condition	645 (61.7%)	453 (57.2%)	192 (75.6%)	< 0.001
Multimorbidity (%)				< 0.001
None	285 (27.3%)	247 (31.2%)	38 (15.0%)	
Moderate	464 (44.4%)	346 (43.7%)	118 (46.5%)	
Complex	297 (28.4%)	199 (25.1%)	98 (38.6%)	
Physical multimorbidity (%)				< 0.001
None	603 (57.7%)	492 (62.1%)	111 (43.7%)	
Moderate	318 (30.4%)	223 (28.2%)	95 (37.4%)	
Complex	125 (12.0%)	77 (9.7%)	48 (18.9%)	
Any primary care (%)	914 (87.4%)	675 (85.2%)	239 (94.1%)	< 0.001
1 or more extended consultations (%)	605/844 (71.7%)	414/611 (67.8%)	191/233 (82.0%)	< 0.001
Median (IQR) UPCI ^a^	0.50 (0.33, 0.70)	0.50 (0.33, 0.67)	0.50 (0.33, 0.71)	0.066
Adequate UPCI (%) ^b^	360/844 (42.7%)	272/611 (44.5%)	88/233 (37.8%)	0.076
Median (IQR) COC ^c^	0.25 (0.10, 0.5)	0.23 (0.11, 0.44)	0.26 (0.10, 0.50)	0.187
Adequate COC (%) ^d^	415/844 (49.2%)	308/611 (50.4%)	107/233 (45.9%)	0.244

^a^Usual Provider Continuity Index (UPCI)

^b^Above cohort median

^c^index of Continuity of Care

^d^Above cohort median;

^e^tests for difference were Chi-square tests for proportions and t-tests for means; Note: total sample size for UPCI and COC analyses was 844 (611 males and 233 females)

### Association between multimorbidity and primary care

Participants with moderate and complex multimorbidity had 2.6 (95%CI 1.9–3.6) and 4.5 (95%CI 3.2–6.4) times the odds of having a high rate of primary care contact (defined as nine or more contacts per person-year), compared to people without multimorbidity, respectively. Of the 256 participants with complex multimorbidity, 48.4 and 40.2% did not have adequate UPCI and COC scores, respectively.

**Fig. 1:**
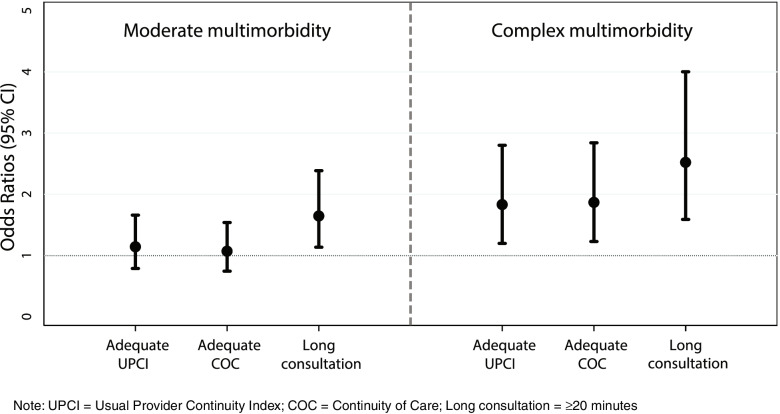
Association between moderate and complex multimorbidity with quality-of-care outcomes, adjusted for model covariates

Table 3 shows the association between multimorbidity and quality of primary care after release from prison. After adjustment for covariates, people with complex multimorbidity were more likely to have adequate UPCI and COC scores, and to have had at least one long consultation, compared to participants with no multimorbidity. However, there was no evidence of an association between moderate multimorbidity and adequate continuity of care (UPCI and COC). Those aged 18–24 years had 36% (95%CI 7–56%) lower odds of having at least one long consultation, compared to those aged 25–44. Indigenous people had nearly 50% lower odds of having adequate continuity of care (UPCI and COC). Figure 1 displays a box plot of odds ratios for the association between multimorbidity and adequate UPCI, adequate COC, and having at least one long consultation, after adjusting for covariates.

**Table 3:** Association between multimorbidity, other covariates, and quality of primary care

	UPCI ≥ 0.5 (***n*** = 837)		COC > 0.25 (***n*** = 840)		Consultation > 20 minutes (***n*** = 840)	
	OR (95%CI)	AOR (95%CI)	OR (95%CI)	AOR (95%CI)	OR (95%CI)	AOR (95%CI)
Multimorbidity						
None	Ref	Ref	Ref	Ref	Ref	Ref
Moderate	1.15 (0.81–1.64)	1.14 (0.79–1.66)	1.11 (0.79–1.56)	1.07 (0.75–1.54)	1.90 (1.33–2.71)	1.64 (1.14–2.39)
Complex	1.84 (1.27–2.68)	1.83 (1.20–2.80)	1.97 (1.36–2.87)	1.87 (1.22–2.84)	3.46 (2.26–5.29)	2.52 (1.59–4.00)
Female	0.76 (0.56–1.03)	0.78 (0.56–1.10)	0.84 (0.62–1.13)	0.91 (0.65–1.28)	2.16 (1.49–3.15)	2.10 (1.41–3.15)
Age in years						
< 25	0.64 (0.45–0.92)	0.68 (0.47–1.00)	0.65 (0.46–0.91)	0.69 (0.48–0.99)	0.54 (0.38–0.77)	0.64 (0.44–0.93)
25–44	Ref	Ref	Ref	Ref	Ref	Ref
45+	2.50 (1.69–3.70)	1.97 (1.30–3.00)	2.70 (1.79–4.06)	2.22 (1.43–3.43)	1.23 (0.79–1.93)	1.24 (0.77–2.02)
Indigenous	0.47 (0.33–0.67)	0.54 (0.37–0.80)	0.48 (0.34–0.68)	0.53 (0.36–0.77)	1.08 (0.74–1.56)	0.91 (0.60–1.37)
Unstable accommodation ^a^	0.59 (0.40–0.86)	0.61 (0.41–0.90)	0.71 (0.49–1.01)	0.73 (0.50–1.07)	1.30 (0.86–1.97)	1.27 (0.82–1.95)
Unemployed ^a^	0.82 (0.63–1.08)	0.92 (0.68–1.24)	0.78 (0.59–1.02)	0.82 (0.61–1.10)	1.46 (1.08–1.98)	1.27 (0.91–1.76)
< 10 years of schooling	0.79 (0.60–1.04)	0.89 (0.65–1.21)	0.88 (0.67–1.16)	0.99 (0.73–1.35)	0.97 (0.72–1.31)	0.87 (0.62–1.22)
Has history of juvenile detention	0.70 (0.51–0.96)	0.84 (0.59–1.20)	0.76 (0.56–1.03)	0.92 (0.65–1.30)	1.20 (0.84–1.69)	1.32 (0.90–1.95)
Prior adult incarceration	0.79 (0.60–1.05)	0.95 (0.69–1.30)	0.82 (0.62–1.09)	0.97 (0.71–1.34)	1.08 (0.79–1.47)	0.88 (0.62–1.24)
Below cohort median level of patient activation (PAM) ^b^	0.64 (0.48–0.84)		0.61 (0.46–0.80)		0.91 (0.68–1.24)	
Released on parole	0.75 (0.59–1.00)		0.74 (0.56–0.99)		0.76 (0.56–1.04)	
Not in a stable relationship	1.02 (0.78–1.35)		1.02 (0.78–1.35)		1.30 (0.96–1.75)	
Transitional program participation	1.02 (0.70–1.47)		0.89 (0.61–1.28)		1.39 (0.90–2.15)	
Ever used injectable drugs	0.87 (0.66–1.15)		0.82 (0.62–1.08)		1.50 (1.11–2.02)	
Below cohort median level of social support (ESSI) ^c^	0.79 (0.60–1.04)		0.81 (0.62–1.06)		1.45 (1.07–1.97)	
Screened positive for intellectual disability (HASI)	1.19 (0.86–1.66)		1.03 (0.74–1.42)		0.92 (0.64–1.31)	
Socioeconomic status of residential area in most disadvantage quintile ^d^	0.89 (0.64–1.22)		0.89 (0.64–1.22)		1.14 (0.80–1.63)	
History of TBI/lead poisoning	0.86 (0.52–1.44)		1.04 (0.63–1.71)		1.15 (0.65–2.04)	
Received service intervention (Passports)	1.02 (0.78–1.34)		1.01 (0.77–1.32)		0.87 (0.65–1.17)	

^a^before prison

^b^PAM median was 63.2

^c^ESSI median was 0.25

^d^Socio-Economic Indexes for Areas (SEIFA). There was no association between receiving the service brokerage intervention and any of the outcomes

Table S3 (Supplementary Material) displays the results of sensitivity analyses. The results of sensitivity analyses were consistent with the primary analysis, with little evidence of substantial changes in estimates when testing study assumptions.

## Discussion

Our study is the first internationally to fully characterise multimorbidity and quantify the association between multimorbidity and quality of primary care in a large cohort of adults released from prison. We found that multimorbidity was present for nearly three quarters of this cohort, which is more than four times the prevalence found in a previous study of the Australian general population [24]. We confirmed previous findings that the health profile of incarcerated women is worse than that of incarcerated men [25].

We found a rate of primary care visits per person-year, after excluding time in prison and censoring for deaths, of 7.33 (95%CI 7.20–7.45) visits per person-year, which is roughly twice the age- and sex-standardised rate for the Queensland population [7]. The health of incarcerated people tends to deteriorate after release from prison [4–6, 26, 27] despite high rates of primary healthcare use after release from custody [7]. In the context of stigma, cultural barriers, poverty, social deprivation, multiple health risk behaviours, and complex health and social needs, adults recently released from prison may receive healthcare that, while more intensive than average, is insufficient to meet their complex health-related needs. For many of these individuals, and particularly for Indigenous people, social determinants of health rather than inadequate access to quality healthcare may be the major contributors to poor health outcomes after release from prison [28].

Our findings suggest that inadequate continuity of care may contribute to the poor health of adults released from prison, especially when multimorbidity is present. In our study, complex multimorbidity was associated with adequate continuity of care, but this was not true for moderate multimorbidity, which includes up to three morbidities. This finding remained after excluding psychiatric morbidity. We also found that Indigenous people were nearly 50% less likely to receive continuous care after release from prison, compared to non-Indigenous people.

Our findings highlight the importance of transitional care clinics, which have become more common in the United States, to improve continuity of care and health outcomes after release from prison [29, 30]. Extended eligibility and access to healthcare through Medicaid is undoubtedly beneficial, but is likely insufficient to improve health outcomes for this population.

We found that multimorbidity (both moderate and complex) was associated with having at least one long consultation within 2 years after release from prison. However, long consultations were 36% less likely for those aged 18–24 years compared to older age groups. Consistent with the literature [31], we also found that the burden of psychiatric illness in this cohort was substantial, especially for females. Previous research has highlighted the benefits of targeted support for people with serious mental illness transitioning from prison to the community [32], given that this group is also typically at higher risk of reincarceration [33].

Our study had four main limitations. First, because people in prison in Australia (as in the United States) are excluded from health insurance, we were unable to measure primary care during periods of reincarceration. Second, due to the limitations of Medicare data, we assessed only three indicators of quality of care. Other markers that would be useful to assess include: provision of preventative healthcare, technical quality of chronic disease management, referrals to allied health and specialist medical services, clinical effectiveness, and patient experience [34]. Third, we were unable to identify instances where patients were visiting multiple doctors in the same clinic, and as such we may have over-estimated continuity of care. Fourth, our data reflected patterns of healthcare use almost a decade ago. However, this remains the only study internationally that has examined linked primary care data in a large, representative, prospective cohort of adults released from prison, and we have no reason to believe that patterns of primary healthcare in this population have changed meaningfully in recent years. Replication of our study in other contexts is warranted, especially in light of the different levels of quality and access to healthcare services for people released from prison in regions and countries worldwide.

## Conclusions

Multimorbidity and mental illness are prevalent in nearly three quarters of adults released from prisons, and are more prevalent in females. In our study, nearly half of the adults released from prison with complex multimorbidity did not receive adequate continuity of care. Younger people (18–24 years) were less likely to have long consultations, and Indigenous people were less likely to receive adequate continuity of care. Our study highlights a missed opportunity to address important health inequities through improved continuity and quality of care for people released from prison, and provides globally relevant evidence of the lack of appropriate healthcare for an already highly marginalised group.

## Supplementary Information

**Additional file 1.**

## Data Availability

Researchers interested in accessing data from the Passports Study should contact Prof Stuart Kinner (s.kinner@unimelb.edu.au) in the first instance. This study combines survey data with administrative data from Queensland Corrective Services (QCS), Medicare Benefits Scheme (MBS), and the National Death Index (NDI) in Australia. Access to these administrative data is contingent on approvals from a number of ethics committees and data custodians.
