# Supplementary material for: Multimorbidity and quality of primary care after release from prison: a prospective data-linkage cohort study
Source: BMC Health Serv Res. 2022 Jul 7;22:876. doi: 10.1186/s12913-022-08209-6 (PMC9264593; doi:10.1186/s12913-022-08209-6)
Supplement: Supplementary file 1 — Additional file 1. [file 12913_2022_8209_MOESM1_ESM.docx]

**SUPPLEMENTARY MATERIAL**

**Table S1. Coding scheme**

| The basis for our scoring system was Miller and Hudon’s CIRS-G^17^ manual which was initially developed for a geriatric primary care setting and contains specific rules for each domain. We created additional coding rules to better capture illnesses that are more common in this young, disadvantaged population, primarily regarding coding of severity in the psychiatric and musculoskeletal domains (which includes skin and dental disease). These included:   - a score of one for skin infections responsive to antibiotics, but a two if multiple courses of antibiotics were necessary; - a score of one for dental disease requiring one or two extractions, but a score of two if three of more extractions were required or there was a high risk of systemic infection; - drug users who experienced withdrawals or had a clear diagnosis of substance use disorder received a score of three, whereas those who had a history of significant drug use or dependence in the past 10 years received a score of two; - current cannabis dependence received a two, and a history of cannabis dependence received a one; - patients with alcohol dependence did not receive a score in the hepatic domain because they receive a score of three in the psychiatric domain instead; - regardless of the cause of psychosis active psychosis received a score of four, psychosis well-managed by antipsychotics received a score of three, and a history of psychosis in the past 10 years that was not currently managed with antipsychotics received a score of two; - a diagnosis of a personality disorder generally received a score of one, unless there was deliberate self-harm or other major concerns.   As per Miller’s guidelines, smoking was scored in the respiratory domain based on pack years smoked (one: 10-19 pack-years, two: 20-40 pack-years, three: >40 pack-years). |
| --- |

**Table S2. Medicare item codes used to define primary care**

| **Short Consultations (<20 mins)** | |
| --- | --- |
| 3  4  13  20  23  24  25  33  35  193  2503  5000  5023  5026 | Level A general practitioner attendance (consulting rooms)  Level A general practitioner attendance (neither at consulting room or aged care facility)  Level A general practitioner attendance (neither at consulting room or aged care facility)  Level A general practitioner attendance (patient accommodated at an aged care facility)  Level B general practitioner attendance (consulting rooms)  Level B general practitioner attendance (neither consulting rooms nor aged care facility)  Level B general practitioner attendance (neither consulting rooms nor aged care facility)  Level B general practitioner attendance (hospital outpatient facility)  Level B general practitioner attendance (at aged care facility)  Level B general practitioner attendance (qualified medical acupuncturist)  Level B general practitioner attendance (cervical smear from under screened person)  Level A general practitioner attendance (consulting rooms)  Level B general practitioner attendance (neither consulting rooms nor aged care facility)  Level A after hours general practitioner attendance |
| **Long Consultations (>20 mins)** | |
| 36  37  38  40  43  44  47  51  197  2700  2701  2713  2715  2717  2559  2725  5043  5046  5067 | Chronic Disease Management Plan Items  Level C general practitioner attendance (neither consulting rooms nor aged care facility)  Level C general practitioner attendance (neither consulting rooms nor aged care facility)  Level C general practitioner attendance (hospital outpatient facility)  Level C general practitioner attendance (aged care facility)  Level D general practitioner attendance (consulting rooms)  Level D general practitioner attendance (neither consulting rooms nor aged care facility)  Level D general practitioner attendance (aged care facility)  Level C general practitioner attendance (qualified medical acupuncturist)  Preparation of primary care mental health plan (>20mins, <40mins)  Preparation of primary care mental health plan (>40mins)  General practitioner attendance in relation to mental health (>20mins)  Preparation of primary care mental health plan (>20mins, <40mins)  Preparation of primary care mental health plan (>40mins)  Level D general practitioner attendance (Asthma Cycle of Care)  General practitioner attendance >40mins providing focused psychological strategies  Level C general practitioner attendance (neither consulting rooms nor aged care facility)  Level C after hours general practitioner attendance  Level D general practitioner attendance (aged care facility) |
| **Unspecified Length** | |
| 1  2  597  599  601  603  725  727  732  735  2712  10987  10989  10993  10994  10996  10997  10998 | Urgent attendance after hours attendance (outside rooms)  Urgent attendance after hours attendance (consulting rooms)  Urgent attendance after hours attendance (consulting rooms)  Urgent attendance after hours attendance (consulting rooms)  Urgent attendance after hours attendance  Urgent attendance after hours (outside consulting rooms)  Review of primary care chronic disease management plan  Review of team care arrangements  Review of team care arrangements or primary care chronic disease management plan  Organisation and coordination of a multidisciplinary case conference  Review of primary care mental health plan  Follow up service provided by practice nurse on behalf of a general practitioner  Wound treatment by Aboriginal and Torres Strait Islander health practitioner on behalf of doctor  Immunisation provided by nurse practitioner on behalf of doctor  Cervical smear and preventative health check provided by nurse practitioner on behalf of doctor  Wound treatment by nurse practitioner on behalf of doctor  Nurse practitioner treatment of chronic disease as part of primary care management plan  Cervical smear provided by nurse practitioner on behalf of doctor |

**Table S3. Association between multimorbidity and quality of primary care; primary and sensitivity analyses**

|  | Unadjusted  (n=844) | Adjusted ^a^  (n=837) | Exclude psychiatric comorbidity  (n=349) | Exclude consultations of unspecified length  (n=775) | Exclude those reincarcerated  (n=504) | Include those with only one primary consultation (n=907)^b^ |
| --- | --- | --- | --- | --- | --- | --- |
| **UPCI ≥0.50** |  |  |  |  |  |  |
| Moderate  Complex | 1.15 (0.81-1.64) 1.84 (1.27-2.69) | 1.19 (0.82-1.72)  1.84 (1.21-2.79) | 1.42 (0.89-2.27)  2.35 (1.12-4.96) | 1.19 (0.82-1.71)  1.10 (0.77-1.57) | 1.06 (0.69-1.63)  2.36 (1.47-3.79) | - |
| **COC >0.25** |  |  |  |  |  |  |
| Moderate  Complex | 1.11 (0.79-1.56) 1.97 (1.36-2.87) | 1.08 (0.75-1.55)  1.79 (1.19-2.68) | 1.31 (0.82-2.08)  3.54 (1.57-8.01) | 1.10 (0.77-1.57)  1.98 (1.35-2.92) | 1.01 (0.67-1.54)  2.53 (1.57-4.10) | - |
| **Extended consultation** |  |  |  |  |  |  |
| Moderate  Complex | 1.90 (1.33-2.71) 3.45 (2.26-5.29) | 1.54 (1.06-2.25)  2.29 (1.44-3.65) | 1.82 (1.11-2.99)  2.30 (0.99-5.35) | 1.85 (1.27-2.67)  3.39 (2.18-5.27) | 2.16 (1.38-3.38)  3.61 (2.06-1.99) | 1.91 (1.37-2.66)  3.53 (2.37-5.23) |

Note: reference category is no multimorbidity. ^a^ adjusted for covariates (see methods). ^b^ This was only calculated for extended consultations, since it was not possible to estimate continuity of care for those with only one primary consultation.
